# Mag-Net Strong Anion Exchange Enables Isolation of Ovarian Cancer Ascites Extracellular Vesicles for Proteomic Biomarker Discovery

**DOI:** 10.21769/BioProtoc.5639

**Published:** 2026-03-20

**Authors:** Tyler T. Cooper

**Affiliations:** 1 Department of Obstétriques and Gynécologie, Université de Montréal, Montréal, QC, Canada; 2 Centre de recherche du Centre hospitalier de l'Université de Montréal (CRCHUM), Montréal, QC, Canada

**Keywords:** Extracellular vesicles, Proteomics, Biofluids, Ascites, Magnetic beads, Strong anion exchange, Cancer

## Abstract

Extracellular vesicles (EVs) are nanoscale particles secreted by all cells and present in all biological fluids, where they carry molecular cargo reflective of health and disease states. Their diagnostic potential is often obscured by the high abundance of non-EV proteins and lipoproteins (e.g., albumin, apolipoproteins) that complicate proteomic analysis of primary biofluids, such as ascites fluid. Conventional isolation strategies face a persistent trade-off between EV purity and yield. To overcome this, a magnetic bead-based protocol (Mag-Net) to enrich EVs according to electrochemical surface charge using strong anion-exchange chemistry (SAX) was adapted for proteomics. Our workflow is specifically adapted to ascites fluid from human or murine sources. This approach effectively separates EVs from high-abundance proteins and lipoproteins, enabling proteomic profiling from as little as 2 μL of ascites fluid. Demonstrated in both murine and human ovarian cancer models, Mag-Net offers a reproducible, scalable, and automation-ready solution for EV isolation from various biofluids.

Key features

• Extracellular vesicles (EVs) from murine and human ascites fluid are effectively enriched using Mag-Net beads.

• EVs are effectively captured and eluted from Mag-Net beads to support Raman spectroscopy, nanoparticle tracking analysis, and atomic force microscopy.

• EV isolation by Mag-Net provides robust proteomic depth obtained by mass spectrometry.

• Robust proteomic data can be obtained from input volumes ranging from 2 to 100 μL of ascites.

## Graphical overview


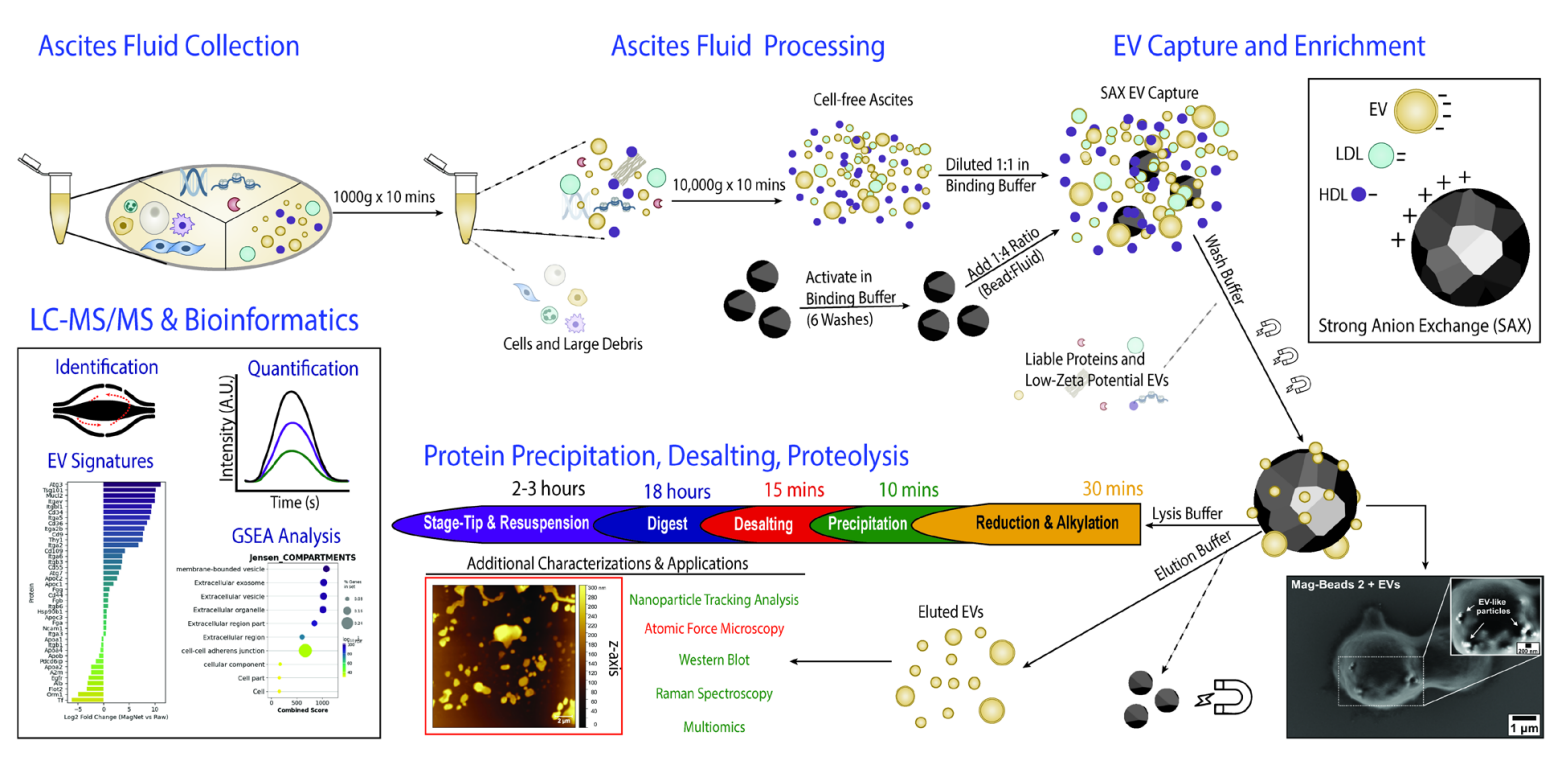


**Isolation of extracellular vesicles from ascites fluid using magnetic beads functionalized with strong anion exchange.** Ascites samples can be collected from human donors or murine models.

## Background

Ascites fluid is a hallmark of several cancers [1], including ovarian cancer [2–4], and represents a valuable yet underutilized source of molecular information [5,6]. Given the absence of reliable biomarkers for early detection, comprehensive proteomic profiling of the tumor microenvironment can help uncover tumor-specific or tumor-associated proteins that reflect disease state and progression [7]. Similar to plasma, ascites is characterized by a high dynamic range of protein abundances. These are derived from diverse cellular sources [1,7,8], posing challenges for in-depth proteomic analysis without depletion of high-abundant species or enrichment of biological sub-compartments. Extracellular vesicles (EVs), which encapsulate molecular cargo representative of their cell of origin, provide a concentrated and disease-relevant snapshot of the tumor milieu [9–11]. Therefore, efficient and selective enrichment of EVs can augment the depth of omics technology and the ultimate success of biomarker discovery efforts [7,12–15].

To address the limitations of conventional EV isolation methods, such as ultracentrifugation (UC) or size-exclusion chromatography (SEC) [12,14–20], we employ a magnetic bead-based approach (Mag-Net) utilizing strong anion-exchange (SAX) chemistry [21]. Mag-Net beads enable scalable input volumes, effective depletion of high-abundance proteins and lipoproteins, and tunable buffer chemistry to exploit EV surface charge (zeta potential) for selective separation [22]. This charge-based isolation strategy is compatible with both manual and automated workflows, and its modular design allows integration with multiomic analyses [23]. The viscosity, lipid, and mucin content of ascites fluid challenge EV isolation [4]; thus, an efficient EV purification from this complex matrix will benefit clinical or diagnostic fields. We have shown that bead-bound EVs isolated from ascites can be divided to support both on-bead proteomic and off-bead EV characterization workflows [23]. Herein, we describe an adaptable protocol optimized for murine and human ascites fluid, enabling efficient EV isolation and proteomic analysis from starting volumes ranging between 2 and 100 μL of input material.

## Materials and reagents

**Biological materials**

1. Murine ascites fluid (ID8 model of ovarian cancer, collected in-house)

2. Human ascites fluid (epithelial ovarian cancer, collected in-house)

**Reagents**

1. MagReSyn^TM^ SAX magnetic beads (ReSyn, catalog number: MR-SAX002)

2. LC–MS-grade water (Fisher Scientific, CAS: 7732-18-5)

3. LC–MS-grade acetonitrile (Fisher Scientific, CAS: 75-05-8)

4. Tris-Bis propane (Sigma-Aldrich, CAS: 64431-96-5)

5. Sodium chloride (NaCl) (Sigma-Aldrich, CAS: 7647-14-5)

6. Urea (Sigma-Aldrich, CAS: 15-37-6)

7. Tris (Sigma-Aldrich, CAS: 77-86-1)

8. N-40 (ThermoFisher, CAS: 9016-45-9)

9. 10 N HCl (Fisher Scientific, CAS: 7647-01-0)

10. Trifluoroacetic acid (TFA) (Sigma-Aldrich, CAS: 76-05-1)

11. Tween-20 (Sigma-Aldrich, CAS: 9005-64-5)

12. n-Dodecyl β-D-maltoside (Sigma-Aldrich, CAS: 69227-93-6)

13. SDS pellets (Sigma-Aldrich, CAS: 151-21-3)

14. TCEP (Thermo Fisher, catalog number: T2556)

15. IAA (Bio-Rad, catalog number: 1632109)

16. Ammonium bicarbonate (Sigma-Aldrich, catalog number: 1066-33-7)

17. Sequencing-grade trypsin (Promega, catalog number: V5113)

18. Sequencing-grade LysC (FujiFilm Bioscience, catalog number: 125-05061)

19. LC–MS-grade 0.1% in water (Fisher Scientific, catalog number: LS1184)

20. UPLC-grade 0.1% in acetonitrile (Fisher Scientific, catalog number: LS1201)

21. C18 Disks (Empore, catalog number: 66883-U)

22. BCA Peptide Quantification kit (Thermo Fisher, catalog number: 23225)

**Solutions**

1. Binding buffer (BB) (see Recipes)

2. Wash buffer (WB) (see Recipes)

3. Elution buffer (EB) (see Recipes)

4. 1% DDM stock (see Recipes)

5. Digestion buffer (DB) (see Recipes)

6. Peptide resuspension solution (see Recipes)

**Recipes**

**1. Binding buffer (BB), pH 6.3**

**Table:** 

Reagent	Final concentration	Quantity or volume
Deionized water (LC–MS grade)	n/a	50 mL
Bis-Tris propane	100 mM	1.412 g
NaCl	150 mM	439 mg
5 N HCl*	to pH 6.3	Estimated 700–750 μL**
Total	n/a	50 mL

*Prepared from 10 N stock. Decreased molarity of HCl prevents polymer leeching from plastics.

**It is important to add in a drop-wise fashion or small volumes (<100 μL). Buffer is basic (>9.5) without titration of HCl.

*Note: Store at room temperature and use within 2 days.*

**2. Wash buffer (WB), pH 6.3**

**Table:** 

Reagent	Final concentration	Quantity or volume
Deionized water (LC–MS grade)	n/a	50 mL
Bis-Tris propane	50 mM	0.706 g
NaCl	150 mM	439 mg
5 N HCl*	to pH 6.3	Estimated 200–300 μL**
Total	n/a	50 mL

*Prepared from 10 N stock. Decreased molarity of HCl prevents polymer leeching from plastics.

**It is important to add in a drop-wise fashion or small volumes (<100 μL). Buffer is basic (>9.5) without titration of HCl.

*Note: Store at room temperature and use within 2 days.*

**3. Elution buffer (EB), pH 6.3**

**Table:** 

Reagent	Final concentration	Quantity or volume
Deionized water (LC–MS grade)	n/a	45 mL
Bis-Tris propane	50 mM	0.706 g
NaCl	1 M	2.92 g
Tween-20 (1%)	0.1%	5 mL
5 N HCl*	to pH 6.3	Estimated 200–300 μL**
Total	n/a	50 mL

*Prepared from 10 N stock. Decreased molarity of HCl prevents polymer leeching from plastics.

**It is important to add in a drop-wise fashion or small volumes (<100 μL). Buffer is basic (>9.5) without titration of HCl.

*Note: Store at room temperature and use within 2 days.*

**4. 1% DDM stock**

**Table:** 

Reagent	Final concentration	Quantity or volume
n-Dodecyl β-D-maltoside stock	n/a	100 mg
Deionized water (LC–MS grade)	n/a	10 mL
Total	1% (w/v)	10 mL

Can be aliquoted and stored at -20 °C for 2 weeks.

**5. Digestion buffer (DB), pH 8.0**

**Table:** 

Reagent	Final concentration	Quantity or volume
Deionized water (LC–MS grade)	n/a	9.8 mL
Ammonium bicarbonate	50 mM	39.5 mg
1% DDM stock	0.02% (w/v)	200 μL
Total	n/a	10 mL

*Note: Store at 4 °C and use within 2 days.*

**Laboratory supplies**

1. 10 μL low-bind pipette tips (Avantor, catalog number: 76322-528)

2. 200 μL low-bind pipette tips (Avantor, catalog number: 76322-150)

3. 1,000 μL low-bind pipette tips (Avantor, catalog number: 76322-154)

4. 2.0 mL round-bottom Eppendorf tubes (Eppendorf, catalog number: 0030108132)

5. 50 mL Falcon tubes (Avantor, catalog number: 525-0610)

6. 15 mL Falcon tubes (Avantor, catalog number: 525-0604)

7. 10 mL stereological pipettes (Corning, catalog number: 4488)

8. 500 mL sterile glass container (Pyrex, Milipore Sigma, catalog number: CLS1000600-6EA)

## Equipment

1. Analytical scale (Metteler-Toledo, model: MA204, Fisher Scientific, catalog number: 01-804-204)

2. Benchtop vortex (OHAS, model: Votrex Genie 2, Fisher Scientific, catalog number: 1-804-423)

3. pH meter (Mettler-Toledo, model: FiveEasy F20, Fisher Scientific, catalog number: 01-912-346)

4. -20 °C freezer

5. -80 °C freezer

6. 4 °C refrigerator

7. Eppendorf^TM^ ThermoMixer^TM^ C (Eppendorf, catalog number: EP5382000023)

8. Magnetic rack (Luna Nanotech, catalog number: MGR-016)

9. UPLC- Vanquish Neo^TM^ (Thermo Fisher)

10. Mass spectrometer Eclipse^TM^ Tribrid Orbitrap (Thermo Fisher)

11. Scanning electron microscopy (Zeiss, model: LEO 1530)

12. Nanoparticle tracking analysis (Particle Metrix, Zetaview)

13. Atomic force microscopy (Bruker, BioScope Catalyst with NCLR-50 Cantilever)

## Software and datasets

1. DIA-NN (v1.9)

2. MSCovert (v3.0)

3. Gwyiddon Nanoscope (v8.5)

4. ZetaView Analyze (v1.4)

5. Zeiss SmartSEM (v6.0)

## Procedure

**A. Activation of SAX magnetic beads**

1. Gently resuspend the stock SAX magnetic bead slurry by gently pipetting the tube several times.

*Note: Use a p200 pipette to avoid spilling solution from the tube. Beads will initially be clustered at the bottom of the tube. Invert the tube to confirm homogenous mixing.*

**Critical:** Immediately reseal the tube with parafilm and store at 4 °C to prevent evaporation.

2. Transfer 100 μL of beads into a 2.0 mL microcentrifuge tube.

*Note: It is critical to bring the tip to the bottom of the Eppendorf to prevent loss of beads on the side of the tube under magnetic force.*

3. Place the tube on a magnetic rack until the solution clears (approximately 30–60 s).

*Note: Beads are black and easy to visualize under a magnet or in solution.*

4. Carefully aspirate the storage buffer by manual pipette and discard without disturbing the bead pellet.

5. Remove the tube from the magnet and add 500 μL of pre-equilibrated binding buffer (BB; see Recipes).

6. Gently resuspend the beads by slow pipetting or by gently tapping/shaking the tube.

*Note: A vortex can be used on the lowest setting possible.*

**Critical:** Avoid vigorous pipetting or vortexing to avoid bead damage or loss.

7. Place the tube back on the magnetic rack and allow the beads to collect.

8. Aspirate and discard the supernatant.

9. Repeat the wash (steps A5–8) for a total of six washes.

10. After the final wash, remove the tube from the magnet and resuspend the beads in 100 μL of fresh BB (i.e., the original bead volume).

**Critical:** Gentle pipetting is necessary, as beads can stick to tube walls when activated (see Figure S1); thus, it is recommended to bring the tip to the bottom of the Eppendorf tube to dispense bead solution.

11. Store the activated beads at 4 °C until use.

**Critical:** Do not place the beads on ice; storage and handling at 4–25 °C is sufficient.

*Notes:*

*1. Properly activated beads should disperse evenly without visible clumps (see Video 1).*

*2. If large clumps are observed, gently break them apart using a pipette tip with minimal pipetting to avoid generating bubbles. Gentle shaking or tapping of the tube can also help to disperse beads.*

**Video 1.**
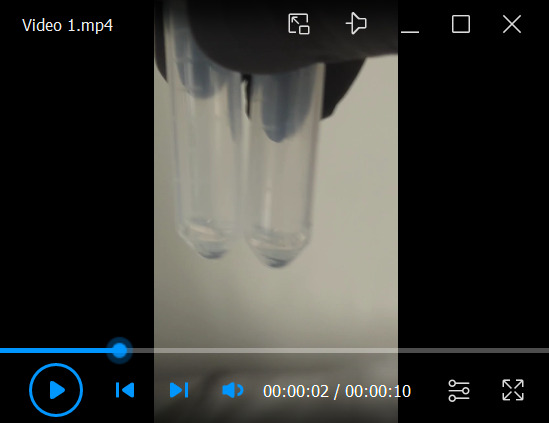
Example of what activated Mag-Net beads look like in binding buffer following manual bead dispersion by gentle shaking. Beads are typically visible by eye, and the absence of bead clumping can be confirmed.

**B. Collection and preparation of ascites fluid**

1. Collect murine ascites by intraperitoneal aspiration using a 1 mL syringe fitted with a 26 G needle, following institutional animal care guidelines. The collected fluid (~0.5–2.0 mL per mouse) is transferred to a sterile 15-mL conical tube and kept on ice until step B4.

2. Collect human ascites according to clinical protocols by accessing the peritoneal cavity under general anesthesia and collecting approximately 50–1,000 mL into a 500 mL sterilized glass container. Transfer 50 mL immediately to a sterile 50-mL conical tube and place on ice.

3. Keep ascites samples on ice and transport them to the laboratory. Process samples within 1–2 h of collection whenever possible.

4. Aliquot 1.8 mL of ascites into a 2.0 mL microcentrifuge tube.

5. Centrifuge the tube at 1,000× *g* for 5 min at 4 °C to pellet cells.

6. Carefully transfer 1.6 mL of the supernatant to a new 2.0 mL microcentrifuge tube without disturbing the pellet.

**Critical:** Avoid disrupting the cell pellet to minimize contamination.

7. If desired, prepare additional aliquots of this clarified supernatant and store at -80 °C for later analysis.

8. Centrifuge the 1.6 mL clarified ascites at 10,000× *g* for 10–20 min at 4 °C to remove cellular debris and large vesicles.

*Note: Depending on the source or composition of ascites, a layer of fat and tissue is often found floating at the top. Avoid pipetting if possible or discard carefully using gentle aspiration to physically push up the wall of the tube.*

9. Transfer 80% of the supernatant to a fresh 2.0 mL microcentrifuge tube. This fraction is referred to as the cell-free ascites.

10. Aliquot the cell-free ascites according to the desired input volume per capture reaction (e.g., 100 μL per reaction).

11. Dilute each aliquot 1:1 (v/v) with BB (e.g., 100 μL ascites + 100 μL BB) and mix by gentle pipetting.

*Notes:*

*1. Minimize repeated freeze–thaw cycles of ascites and cell-free ascites, as these can disrupt vesicles.*

*2. Volumes or sample numbers can be scaled proportionally depending on the available ascites volume, magnetic rack availability, and pipetting capabilities. We tend to manually process 12–24 samples per protocol. Robotic liquid handlers can increase sample throughput.*

**C. SAX capture and enrichment of EVs**

1. Equilibrate the activated SAX beads (section A) and diluted cell-free ascites (section B) to room temperature.

2. For 100 μL of cell-free ascites diluted 1:1 with BB for a total volume of 200 μL, add 25 μL of activated SAX beads for a final ratio of 1:4 (bead to input volume of cell-free ascites).

*Notes:*

*1. Bead volumes are calculated based on input volumes of cell-free ascites (i.e., 25 μL of beads for 100 μL of ascites). However, cell-free ascites is diluted 1:1 with BB before the beads are added.*

*2. Volumes of BB and beads will need to be adjusted based on the starting volume of ascites fluid. Proteomic data can be obtained from as little as 2 μL of input volume.*

**Critical:** Optimization of the ratio is required depending on the input volume. For example, starting ascites volumes under 10 μL were incubated at a 1:2 or 1:3 ratio.

3. Gently mix the suspension by gentle shaking or using a Thermomixer set to 600 rpm for 2 min.

4. Incubate the mixture at room temperature for 10–15 min with gentle agitation to allow EV binding to the SAX beads.

*Note: A thermomixer set to 600 rpm was sufficient to keep beads in suspension without damaging EVs.*

5. Place the tube on a magnetic rack for 2 min until the solution is clear of beads.

6. Carefully collect and discard the unbound supernatant. Alternatively, the supernatant can be retained to assess EV capture efficiency or to isolate secondary EV subpopulations.

**Critical:** Avoid disturbing the bead pellet when removing the supernatant. Pipette from the opposite side of the tube relative to the direction of magnetism.

*Note: The supernatant can be retained for assessing depletion efficiencies or secondary EV enrichments.*

7. Remove the tube from the magnet and add 500 μL of WB.

*Note: Wash volume should be adjusted based on initial input volume. We recommend washing with 5× the volume of the initial input volume or 50 μL total for input volumes less than 10 μL.*

8. Gently resuspend the bead–EV complex by gentle agitation for 2 min.

*Note: A thermomixer set to 600 rpm was sufficient to keep beads in suspension without damaging EVs.*

9. Place the tube back on the magnetic rack and allow the beads to collect for 2 min; then, discard the wash supernatant.

10. Repeat the wash (steps C7–9) for a total of three washes.

*Notes:*

*1. Minimize pipetting and vigorous mixing once EVs are bound to the beads to reduce sample loss.*

*2. Maintain consistent incubation and wash times across sample specimens to ensure comparability.*

*3. Supernatants from binding and wash steps can be retained to monitor depletion of abundant proteins or EVs.*

*4. Additional washes with a magnet can be included if albumin or lipoprotein contamination remains elevated.*

11. After the final wash, carefully remove as much residual buffer as possible without disrupting the bead pellet.

12. Proceed to section D for on-bead protein extraction, proteolysis, and peptide cleanup, or to section E for elution of intact EVs for additional characterization, such as nanoparticle tracking analysis.

**D. Protein precipitation, proteolysis, and LC–MS/MS**

1. Resuspend the SAX bead–EV pellet in 50–55 μL of lysis buffer (e.g., 4 M urea, 50 mM Tris, pH 8.0, 150 mM NaCl, 2% SDS, 1% NP-40).

2. Gently mix by pipetting or brief low-speed vortexing to fully resuspend the beads.

**Critical:** Do not sonicate.

3. Incubate at 24 °C for 15 min with gentle shaking to lyse EVs and solubilize proteins.

*Note: A thermomixer set to 600–800 rpm was sufficient to keep beads in suspension.*

4. (Optional) Remove 5 μL of sample for protein quantification using BCA.

5. Add TCEP to a final concentration of 5–10 mM.

6. Add IAA to a final concentration of 30 mM and incubate in the dark at room temperature for 30 min to alkylate cysteines.

*Note: Incubation can occur on a thermomixer by covering the top with aluminum foil. Set the thermomixer to 24 °C and 800 rpm.*

7. Add acetonitrile to the lysis buffer to obtain 70% acetonitrile. For example, 117 μL of acetonitrile is added to 50 μL of lysis buffer to reach a final concentration of 70% acetonitrile.

8. Incubate for 15 min on the thermomixer set to 24 °C and 800 rpm.

9. Place samples on the magnetic rack and wait 2 min before removing the supernatant.

10. Gently add 200 μL of 80% EtOH while keeping the sample magnetized and incubate for 2 min.

**Critical:** It is important not to disrupt the bead pellet. Thus, we recommend slowly pipetting to the wall of the tube opposite the magnetic interface.

11. Carefully aspirate and discard supernatant.

12. Repeat steps D9–10 two more times, for a total of 3 washes.

13. Gently add 200 μL of 95% acetonitrile while keeping the sample magnetized and incubate for 2 min.

14. Carefully aspirate and discard the supernatant.

15. Remove samples from the magnet.

16. Add 100 μL of DB directly to the bead pellet.

*Notes:*

*1. Pipetting DB on the pellet will facilitate dispersal of beads into solution.*

*2. Gentle shaking or brief water bath sonication (<3 s) can help to disperse beads.*

17. Add sequencing-grade LysC (and/or other proteases) at an enzyme-to-protein ratio of 1:100 (w/w).

18. Incubate the digestion mixture at 37 °C for 2 h on the thermomixer set to 900 rpm.

*Note: RPM can be increased if beads are not remaining in solution; however, avoid excessive splashing on the side of the tubes.*

19. Add sequencing-grade trypsin (and/or other proteases) at an enzyme-to-protein ratio of 1:25 to 1:50 (w/w).

*Note: Digestion is the most time-consuming step of the protocol (Table 1) and can be adapted to more rapid digestion protocols using alternative enzymes or proteolytic workflows.*

**Table 1.** Summary of EV isolation and analysis by LC–MS/MS

Step/assay	Tubes used	Time of step/assay	Temperature
Ascites collection*	15 or 50 mL conical	TBD*	Stored on ice or 4 °C
Removal of cells and debris	2.0 mL Eppendorf	30 min	4 °C
Activation of beads	2.0 mL Eppendorf	5 min	RT
EV capture	2.0 mL Eppendorf	10 min	RT
EV washing	2.0 mL Eppendorf	15 min	RT
Option 1: Elution	2.0 mL Eppendorf	15 min	RT
Option 2: Lysis	2.0 mL Eppendorf	10 min	RT
Reduction/alkylation	2.0 mL Eppendorf	30 min	RT
Protein precipitation	2.0 mL Eppendorf	15 min	RT
Protein cleanup	2.0 mL Eppendorf	10 min	RT
Protein digestion	2.0 mL Eppendorf	18 h**	37 °C**
Peptide desalting	1.5 mL Eppendorf	1 h	RT
LC–MS/MS and analysis	1.5 mL Eppendorf	1 h	n/a
Total time	n/a	~22.5 h	n/a

*Time of collection is dependent on the institution and protocol.

**Enzymes and digestion times can be modified.

20. Incubate the digestion mixture at 37 °C for 12–16 h on the thermomixer set to 900 rpm.

*Note: RPM can be increased if beads are not remaining in solution; however, avoid excessive splashing on the side of the tubes.*

21. Separate the digest from beads and terminate the digestion by acidifying the sample to pH < 3 using 10% TFA (final concentration 1%–2%).

*Note: An additional wash with LC-MS grade water or DB can help to recover residual peptides.*

**Critical:** Do not acidify samples with beads present can increase the risk of polymer contamination and poor MS results.

22. Centrifuge at 16,000× *g* for 10 min at room temperature to remove any insoluble material and transfer the supernatant containing peptides to a new 1.5-mL microcentrifuge tube.

23. Desalt peptides using C18 StageTips or solid-phase extraction cartridges according to the manufacturer’s instructions.

**Critical:** Samples obtained from less than 10 μL of starting ascites volume are not subjected to peptide clean-up to avoid sample loss. In these cases, proceed to step D25.

24. Elute peptides with 60% acetonitrile containing 0.1% formic acid.

25. Dry peptides eluted in step D23 or peptides from digests in step D22 in a vacuum concentrator until less than 10 μL remains.

26. Reconstitute dried peptides in 0.1% formic acid with 0.01% DDM (e.g., 10–100 μL) for LC–MS/MS analysis.

27. Sample 50–500 ng of peptides by LC–MS/MS operating in data independent acquisition using a gas-phase fractionation strategy (GPF-DIA) [24].

**Critical:** Samples obtained from less than 10 μL of starting ascites volume typically require FAIMS or alternative ion mobility to obtain the sensitivity needed.

*Notes:*

*1. Peptide concentrations can be determined using BCA, Bradford, or nanodrop workflows.*

*2. Avoid over-drying peptide samples, as this can decrease solubility during reconstitution.*

**E. EV elution for additional characterizations (optional)**

1. Following SAX capture and washing (step C11), resuspend the bead–EV pellet in 100–200 μL of EB.

2. Gently mix the suspension by slow pipetting to avoid bead loss and EV disruption.

*Notes:*

*1. As EVs are eluted, beads will disperse into the solution.*

*2. Manual shaking of the tube is often sufficient to induce bead dispersion.*

**Critical:** Avoid excessive pipetting to limit EV disruption and sample loss.

3. Incubate at room temperature for 10–15 min with gentle agitation on the thermomixer set at 400 rpm.

4. Place the tube on the magnetic rack and allow the beads to separate from the eluted EVs.

5. Carefully transfer the EV-containing supernatant to a new low-protein-binding microcentrifuge tube.

6. (Optional) Repeat the elution once more with fresh elution buffer and pool the eluates.

**Critical:** Proceed with caution, as increased volumes will dilute EV concentrations beyond detectable levels.

7. Adjust the buffer composition according to downstream applications:

a. For nanoparticle tracking analysis (NTA), dilute EVs in particle-free PBS.

b. For western blotting, add appropriate sample buffer and denature/reduce as required.

c. For electron microscopy, process according to EM sample preparation protocols.

*Note: 10 or 100 kDa ultrafiltration microcentrifuge tubes can be used for buffer exchange.*

8. Store eluted EVs on ice for short-term use or at -80 °C for long-term storage.

**Critical:** Minimize freeze–thaw cycles to preserve EV integrity.

## Data analysis

**Figure 1.**
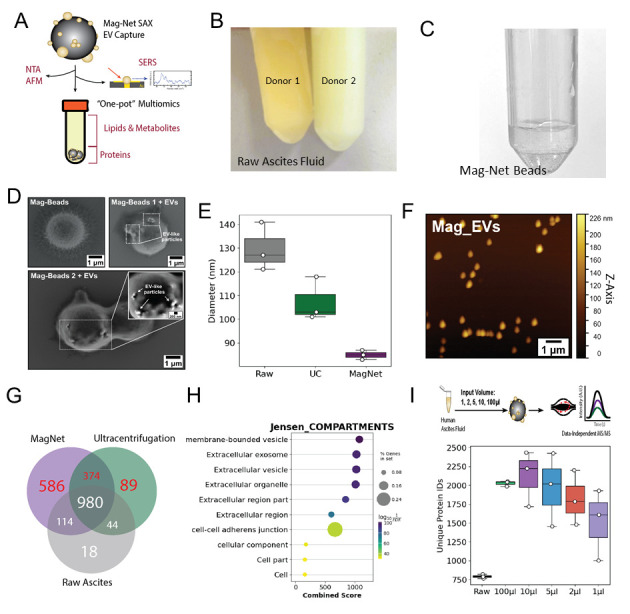
Capture, elution, and proteomic analysis of EVs using strong anion exchange (SAX) magnetic beads. (A) Schematic of EV capture from ascites using Mag-Net SAX magnetic beads and examples of downstream applications, including mass spectrometry for multiomics, biophysical analyses, and surface-enhanced Raman spectroscopy single-EV analysis. (B) Representative photograph of two human ascites samples showing differences in color and clarity. Ascites on the left is from a donor with FIGO II mucinous ovarian cancer, and ascites on the right is from a donor with FIGO III high-grade serous carcinoma. (C) Mag-Net beads resuspended in binding buffer (pH 6.3). (D) Scanning electron microscopy images of Mag-Net beads before and after incubation with ascites fluid. EV-like particles are visible on the bead surface after incubation. Scale bars = 1 μm. (E) Nanoparticle tracking analysis of raw ascites, EVs isolated by ultracentrifugation (UC), and EVs isolated using Mag-Net beads. (F) Atomic force microscopy images of EVs eluted from Mag-Net beads. Scale bar = 1 μm. (G) Venn diagram comparing EV proteomes from Mag-Net-isolated EVs, UC-isolated EVs, and cell-depleted ascites. Mag-Net identified an additional 586 compared to UC; however, a share of 374 proteins was not detected in cell-free ascites. (H) The combination of unique proteins in Figure 1G (red) was analyzed by Jensen Compartments enrichment analysis, in return validating an enrichment of EVs using both methods. (I). The minimum input volume needed to maintain EV proteomic signatures was 2 μL, compared to the standard 100 μL and raw cell-free ascites. Although more proteins were identified in 1 μL than raw ascites, a depletion of classic EV markers relative to 2 μL was observed (refer to the research article [23]). Abbreviations: NTA = nanoparticle tracking analysis; AFM = atomic force microscopy; nFC = nanoflow cytometry; SERS = surface-enhanced Raman spectroscopy. EVs = extracellular vesicles.

Raw LC–MS/MS files were converted to mzML files using msconvert with the following parameters: peakPicking:msLevel =1-, zeroSamples:removeExtra 1-. Downstream missing value imputation, enrichment analysis, and data visualization were performed in Python 3.13, using libraries including MissForest [25] and GSEApy [26]. Proteomic signatures of raw, UC EVs, or Mag-Net EVs were generated using the venn library in Python (Figure 1G). Jesen Compartment Enrichment Analysis, shown in Figure 1H, was performed using the GSEApy library after filtering for proteins exclusively detected in UC and/or Mag-Net EV proteomes. Using label-free quantification values, the number of identified proteins is presented for various input volumes of ascites fluid from the same donor, repeated in technical triplicates (Figure 1I).

## Validation of protocol

This protocol has been used and validated in the following research article:

• Cooper et al. [23]. Isolation of Extracellular Vesicles from Minimal Volume Ascites Fluid Using Strong Anion Exchange Magnetic Beads. *bioRxiv*, 2025-09.

## General notes and troubleshooting

**General notes**

1. Ascites fluid color, clarity, and protein concentration can vary considerably between donors. Protein quantification is performed for each donor using a raw sample and precipitated protein resuspended in lysis buffer.

2. Thermomixers from various vendors or variations in the size of tube holders on the mixer can affect bead dispersion; thus, it is recommended to test RPM with beads in binding buffer prior to the start of experimentation. This can help to avoid loss of beads on the side of the tube (Figure S1 and Video 1).

3. Combining isolation techniques, such as UC followed by Mag-Net or Mag-Net followed by UC, did not increase the sensitivity or depth of proteomic analyses in our hands. Classical EV markers were elevated with the combined technique. In practical use, Mag-Net is able to effectively enrich EVs from low volumes of input material, relative to common isolation techniques; however, persistent contamination of proteins associated with high-density lipoproteins and ribosome translational complexes needs to be further explored.

4. All mass spectrometry–based analysis should use cell-free ascites as an internal control to confirm 1) EV enrichment, 2) baseline dynamic range of biofluid, and 3) identification of highly abundant proteins specific to the biofluid and donor.

5. While this protocol was written for the procedure to be performed manually and at a low scale (maximum 24 samples per day), the inclusion of robotic liquid handlers equipped with magnets and mass spectrometry instrumentation with increased sensitivity and speed would greatly improve the high-throughput automation of this protocol.

**Troubleshooting**

**Problem 1:** Aggregation of beads following EV capture.

Possible causes: Residual cells, cellular debris, or viscosity in the ascites fluid.

Solutions: If using samples thawed after freezing, it is recommended to centrifuge at 10,000× *g* for 5 min prior to step C2. Starting the protocol with a more diluted sample can help avoid bead aggregation. Likewise, adjustments to the bead ratio to the starting volume may need to be made.

**Problem 2:** Low EV binding (chemical).

Possible causes: Improper pH of binding buffer or vigorous mixing.

Solutions: Recalibrate the pH meter. If the problem persists, try to modify the buffer in 0.2 pH increments.

**Problem 3:** Low EV binding (physical).

Possible cause: Vigorous mixing.

Solutions: Try to reduce the RPM on the mixer, avoid pipetting, and manually disperse beads by gently shaking the tube (see Video 1).

**Problem 4:** Low protein identification on LC–MS/MS.

Possible causes: Inefficient protein precipitation, digestion, and/or C18 cleanup.

Solutions: Issues with protein precipitation can be addressed by using fresh acetonitrile or increasing incubation times up to 20 min. Additional protein washes (80% ethanol or 95% acetonitrile) may also help improve precipitation and digestion efficiency. Issues with digestion efficiency can be addressed by using fresh buffers, enzymes, and optimizing mixing (800–1,300 rpm). Issues with peptide recovery during C18 cleanup can be attributed to improperly activated C18 or inefficient elution from the C18 matrix. Ensure all stage-tip buffers are made fresh daily for maximum performance.

## Supplementary information

The following supporting information can be downloaded here:

1. Figure S1. Example of Mag-Net beads sticking to the walls of the tube following activation. Gentle manual bead dispersion (see Video 1) can sometimes recover these beads.
